# TGM2 regulated by transcription factor NR3C1 drives p38 MAPK-mediated tumor progression and immune evasion in lung squamous cell carcinoma

**DOI:** 10.3389/fimmu.2025.1595907

**Published:** 2025-09-18

**Authors:** Chunlong Lin, Shangfu Li, Liang Yi, Huiling Zhou, Zhiyi Xiao, Zhuo Yang, Qingping Chen, Xiangyang Peng, Kanao Li, Qing Wang, Wei Liu, Ni Li, Lun Li, Duanlin Du, Qi Xu, Lingge Yang

**Affiliations:** ^1^ Department of Respiratory and Critical Care Medicine, Yueyang People’s Hospital of Hunan Normal University, Yueyang, China; ^2^ Department of Oncology, Yueyang People’s Hospital of Hunan Normal University, Yueyang, China; ^3^ Yueyang Municipal Key Laboratory of Molecular Biology for Respiratory Oncology, Yueyang, China; ^4^ Department of Pathology, Yueyang People’s Hospital of Hunan Normal University, Yueyang, China

**Keywords:** lung squamous cell carcinoma, TGM2, p38 MAPK signaling, immune evasion, tumor microenvironment, NR3C1

## Abstract

**Background:**

Lung squamous cell carcinoma (LUSC) is highly malignant with limited therapeutic targets. Prior studies indicate transglutaminase 2 (TGM2) regulates the tumor microenvironment, but its mechanisms in driving LUSC progression and immune evasion remain unclear.

**Methods:**

The prognostic value of TGM2 was analyzed using the TCGA LUSC cohort. Key genes were screened *via* random forest algorithm. Functional validation was performed in NCI-H520 and SK-MES-1 cell lines. Proteomics, GSEA, and TIMER2.0 assessed downstream pathways and immune infiltration. Transcriptional regulatory databases predicted the upstream transcription factors of TGM2, which was then validated by chromatin immunoprecipitation (ChIP)-qPCR.

**Results:**

TGM2 was an independent prognostic factor for LUSC. High TGM2 expression correlated with reduced overall survival (OS, P = 0.00018) and disease-free survival (DFS, P = 0.00019). TGM2 promoted proliferation, migration, invasion, clonogenicity, and suppressed apoptosis in LUSC cells by activating p38 MAPK signaling. Elevated TGM2 levels were associated with an immunosuppressive microenvironment: decreased Th1 (R = -0.186, P < 0.0001) and NK cell infiltration (R = -0.116, P = 0.0092), and increased M2 macrophage (R = 0.164–0.528, P < 0.0001) and cancer-associated fibroblast infiltration (R = 0.469, P < 0.0001). NR3C1 was identified as a key transcription factor regulating TGM2. ChIP-qPCR analysis confirmed that NR3C1 binds to a specific site (921-935) within the TGM2 promoter, and their expression showed a strong positive correlation (R = 0.53, P < 0.0001).

**Conclusion:**

TGM2 drives LUSC progression *via* p38 MAPK activation and shapes an immunosuppressive microenvironment, which is transcriptionally regulated by NR3C1. This study supports TGM2 as a prognostic biomarker and suggests its potential as a therapeutic target, which may inform future combination immunotherapy strategies.

## 1 Introduction

Lung cancer remains one of the most common and deadly cancers globally, posing a significant threat to public health. According to the latest data from the International Agency for Research on Cancer, although the estimated incidence of lung cancer has decreased, it remains the leading cause of cancer-related mortality worldwide (1). Lung squamous cell carcinoma (LUSC), the second most common of lung cancer after lung adenocarcinoma (LUAD), constitutes approximately 30% of all cases (2). Due to its subtle onset and low detection rate rates in the early stages, a large proportion of patients with LUSC are diagnosed at advanced stages, missing the opportunity for curative surgery (3). Even among those who undergo surgery, the 5-year survival rate remains bleak, at only 12.4% (4). Unlike LUAD, LUSC is characterized by fewer gene mutations but greater tumor heterogeneity, limiting the effectiveness of mutation-targeted therapies (5, 6). Moreover, treatment options such as chemotherapy and radiotherapy offer limited long-term survival benefits, contributing to a generally poor prognosis (7).

Current treatment modalities for lung cancer include surgery, radiotherapy, chemotherapy, targeted therapy, and immunotherapy. Among these, immunotherapy has transformed oncology by providing a favorable safety profile, inducing immune memory for sustained efficacy, and demonstrating significant effectiveness across diverse patient populations (8). For metastatic non-small cell lung cancer (NSCLC), the introduction of immune checkpoint inhibitors (ICIs) targeting programmed death-1 (PD-1), programmed death ligand 1 (PD-L1), and cytotoxic T-lymphocyte-associated protein 4 (CTLA-4) has markedly improved survival rates in both first-line and second-line treatments over the past decade (9). Immunotherapy offers promising new novel therapeutic options for long-term survival in patients with NSCLC and has fundamentally altered the treatment landscape. However, the efficacy of ICIs varies greatly between tumor types, with only approximately 20% (10–12) of patients demonstrating active T-cell responses and benefiting clinically. Therefore, identifying reliable biomarkers to predict patient prognosis and response to immunotherapy is critical for advancing precision medicine and improving treatment outcomes.

In preliminary research, a predictive model based on tumor immune cell infiltration within the tumor microenvironment (TME) was developed to assess immunotherapy efficacy and differentiate “cold” from “hot” tumors in patients with LUSC (13). Using LUSC datasets from one training cohort and three independent validation cohorts, a 17-gene immune phenotype scoring model was established, effectively stratifying patients into high and low immune infiltration subgroups. The high-infiltration subgroup demonstrated significant enrichment of immune-related signaling pathways, suggesting that molecular subtyping based on TME immune infiltration offers valuable predictive insights into immunotherapy response and potential prognostic implications in LUSC.

Random forest algorithms combined with Cox regression analyses identified Transglutaminase 2 (TGM2) as the top prognostic gene among the 17 candidates. Its independent prognostic utility was further validated in the TCGA-LUSC cohort. TGM2, a multifunctional protein, plays key roles in tumor autophagy, metastasis, drug resistance, and pro-tumor inflammation (14). Its overexpression correlates strongly with drug resistance and metastatic phenotypes in various cancers (15–19), primarily through autophagy inhibition (20, 21). As a critical promoter of epithelial-mesenchymal transition (EMT), TGM2 facilitates metastasis by activating FAK, AKT, and NF-κB signaling pathways (22). TGF-β-induced EMT in mammary epithelial cells depends on TGM2 expression (23). Additionally, TGM2 regulates both innate and adaptive immunity by inhibiting T/B cell proliferation and activation while promoting myeloid-derived suppressor cell (MDSC) infiltration (14). TGM2-deficient mice exhibit impaired T cell activation and a reduced the proportion of MDSC/tumor-associated macrophage (TAM) cells, significantly enhancing anti-tumor immunity within the TME (24). These properties position TGM2 as a novel immunoregulatory target with promising therapeutic potential (25).

Recent studies by Chang et al. (26) have emphasized the clinical significance of TGM2 in LUSC, linking it to poor prognosis, tumor-promoting inflammation, and potential implications for immunotherapy response. However, the precise molecular mechanisms by which TGM2 drives LUSC progression and shapes the TME, particularly its downstream signaling effectors and upstream regulatory factors, remain incompletely understood. Building on previous research, this study aims to further investigate the specific impact of TGM2 on LUCS progression through experimental validation and integrated bioinformatics analysis, with the goal of uncovering key regulatory mechanisms and validating its functional roles. By elucidating the role of TGM2 in promoting tumor development, shaping the immunosuppressive TME, and exploring its interactions with upstream transcription factors (TFs), this work aims to provide innovative strategies and therapeutic targets for precision targeted therapy, combination immunotherapy, and prognostic evaluation in lung cancer. These advancements are expected to drive progress in lung cancer diagnosis and treatment.

## 2 Materials and methods

### 2.1 Data sources and processing

This study integrated multi-omics data with experimental validation. Publicly available datasets used included: (1) The Cancer Genome Atlas (TCGA)-LUSC Cohort (https://cancergenome.nih.gov): RNA sequencing (RNA-seq) data, clinical survival records, and proteomic profiles of patients with LUSC were retrieved from the TCGA database using the TCGAbiolinks package (v2.28.4) in R 4.3.1 (27). Data were collected on August 21, 2024, using the GDCquery function with the following filters: project = “TCGA-LUSC”, data.category = “Transcriptome Profiling”, data.type = “Gene Expression Quantification”, and workflow.type = “STAR - Counts”. Genes with zero counts in over 50% of samples were excluded during quality control. Raw counts were normalized to transcripts per million (TPM) using trimmed mean of M-values (TMM) scaling *via* the edgeR::calcNormFactors function, followed by log2 transformation. Batch effects from technical confounders were corrected with the ComBat algorithm. The final processed matrix was validated for robustness in downstream analyses. A total of 496 samples with complete clinical data and transcriptomic data, along with 322 samples with proteomic profiles, were included. (2) GDSC Database (https://www.cancerrxgene.org) (28): TGM2 expression levels in LUSC cell lines were screened through this database. The NCI-H520 (low TGM2 expression) and SK-MES-1 (high TGM2 expression) cell lines were selected for functional experiments.(3) UALCAN Database (https://ualcan.path.uab.edu) (29): This platform was used to identify signaling pathways potentially influenced by variations in TGM2 protein expression levels. (4) TIMER 2.0 Database (http://timer.cistrome.org/) (30): This tool enabled analysis of to analyze immune cell infiltration within the samples.

### 2.2 Gene screening and validation

Using the immune phenotype scoring model previously developed, expression profiles of the 17 genes and patient survival data were integrated. Random forest modeling was performed with the randomForestSRC package (version 3.2.3), using parameters ntree = 1000 and nodesize = 15, to assess the significance of these genes in predicting overall survival (OS) and disease-free survival (DFS). TGM2, identified as the gene with the highest prognostic significance, was selected for further analysis. Survival analysis was conducted with the survival package (version 3.5-7), and survival curves were generated with the survminer package (version 0.4.9). The optimal cutoff value for OS (TPM = 210.89) was determined using the surv_cutpoint function, based on survival-specific maximally selected rank statistics, which accounts for to respect the time-to-event nature of OS data, classifying patients into high-risk (n = 51) and low-risk (n = 444) groups. Multivariate Cox proportional hazards regression analysis confirmed the independent prognostic value of TGM2, and the results were visualized using the forestploter package (version 1.1.2).

### 2.3 Cell culture and genetic manipulation

Cell Lines and Culture Conditions: Human LUSC cell lines NCI-H520 and SK-MES-1 (Shanghai QuiCell Biotechnology Co., Ltd.) were cultured in RPMI-1640 medium supplemented with 10% fetal bovine serum (FBS) and 1% penicillin-streptomycin at 37°C under 5% CO_2_.

TGM2 Overexpression and Knockdown: Lentiviral vectors were transfected into NCI-H520 cells to establish stable TGM2-overexpressing (TGM2-OE) cell lines. Empty vector and untreated cells served as negative controls (NC) and blank controls (Ctrl), respectively. SK-MES-1 cells were transfected with three specific siRNAs (siRNA-1, siRNA-2, and siRNA-3), with NC and Ctrl used as controls. Transfection efficiency was validated by quantitative Reverse Transcription Polymerase Chain Reaction (qRT-PCR) under the following conditions: 95°C for 3 min (initial denaturation); 40 cycles of 95°C for 15 sec, 60°C for 30 sec, 72°C for 30 sec, followed by melt curve analysis (95°C for 15 sec, 60°C for 1 min, 95°C for 15 sec) to verify amplification specificity, and Western blot analysis. Primer and siRNA sequences are listed in **Supplementary Table S1**. Primary antibodies were diluted as follows: anti-TGM2 (HUABIO, ET1706-35; 1:1000), anti-p38 MAPK (HUABIO, HA722036; 1:1000), anti-Phospho-p38 MAPK (T180/Y182) (HUABIO, ER1903-01; 1:1000), anti-NR3C1 (Cell Signaling Technology, #12041; 1:1000), anti-GAPDH (Proteintech, 60004-1-Ig; 1:5000), anti-β-tubulin (CST, #2146; 1:1000). HRP-conjugated secondary antibodies (Cell Signaling Technology, #7074 for rabbit, #7076 for mouse) were used at a 1:10,000 dilution.

### 2.4 Cellular phenotypic assays

Cell Proliferation Assay: Cell proliferation was assessed using a CCK-8 kit (Shanghai Sangon Biotech Co., Ltd.) at 0, 24, 48, 72, and 96 hours post-transfection by measuring absorbance at 450 nm. Proliferation curves were generated accordingly.

Migration and Invasion Assays: (1) Wound Healing Assay: Cells were seeded in 6-well plates to form a monolayer. A scratch was made using a sterile pipette tip, and wound closure was observed after 24 hours. (2) Transwell Assay: For migration assays, 2×10^4^ cells/well in serum-free medium were seeded into the upper chamber of uncoated 24-well Transwell plates. For invasion assays, 2×10^4^ cells/well in serum-free medium were seeded into Matrigel-coated chambers. After incubation, cells that migrated through the membrane were fixed, stained, and counted.

Colony Formation Assay: Cells (1,000 per dish) were cultured in 6 cm dishes for 10 days, followed by crystal violet (Shanghai Beyotime Biotechnology Co., Ltd.) staining was performed after fixation. Colonies were counted manually.

Apoptosis Detection: Apoptosis rates were assessed using an Annexin V-APC/7-AAD apoptosis detection kit (Wuhan Elabscience Biotechnology Co., Ltd.) by flow cytometry.

### 2.5 Proteomic analysis and signaling pathway validation

Reverse-Phase Protein Array (RPPA) Analysis: RPPA data from TCGA-LUSC samples were downloaded to identify differentially expressed proteins (DEPs) between TGM2 high-risk and low-risk groups. Correlation analysis was performed to identify proteins significantly associated with TGM2 expression.

Pathway Screening: The UALCAN database was used to identify key cancer-related signaling pathways potentially influenced by TGM2 expression.

Pathway Validation: Western blotting was performed to detect key proteins and their phosphorylation levels identified in RPPA and pathway screening analyses, thereby validating the regulatory role of TGM2 in these pathways.

### 2.6 Immune-related pathway enrichment and immune cell infiltration analysis

Differentially Expressed Genes (DEGs) Screening: DEGs between TGM2 high-risk and low-risk groups were identified using the limma package (version 3.56.2) (31), DEGs between TGM2 high-risk and low-risk groups were identified with thresholds set at |Fold Change (FC)| ≥2 and false discovery rate (FDR) <0.05. Results were visualized using the ggVolcano package (version 0.0.2).

Gene Set Enrichment Analysis (GSEA): The clusterProfiler package (version 4.8.3) (32) was used to analyze Kyoto Encyclopedia of Genes and Genomes (KEGG) pathway enrichment of DEGs *via* the gseKEGG function, which directly accesses the KEGG pathway database (https://www.kegg.jp) through its application programming interface (API) at the time of access (9^th^ September 2024). Enrichment plots were generated using the GseaVis (version 0.0.9) and enrichplot (version 1.20.3) packages.

Immune Infiltration Analysis: Transcriptomic data from TCGA-LUSC were uploaded to TIMER2.0 with species set to “human” and cancer type to “LUSC”. Immune cell infiltration scores were obtained and correlated with TGM2 TPM values. Statistically significant results (P < 0.05) were selected for presentation.

### 2.7 Exploration of transcriptional regulatory factors

Potential transcriptional regulators of TGM2 were predicted by integrating data from two databases: (1) Cistrome DB Toolkit (http://cistrome.org/db/#/): Species = “Human hg38”, gene symbol = “TGM2”, data type = “Transcription factor/chromatin regulator”, half-decay distance to TSS = 10 kb. (2) hTFtarget (https://guolab.wchscu.cn/hTFtarget/#!/): Direct search for ‘TGM2’ in the Quick Search module. Overlapping TFs between both databases and DEGs were prioritized for further analysis. A heatmap was generated to display TFs based on TGM2 risk groups. The top-ranked TF, NR3C1, was selected for additional investigation: (1) Expression Correlation: The GEPIA database (http://gepia.cancer-pku.cn) (33) was used to analyze the correlation between NR3C1 and TGM2 expression in LUSC. (2) Group Differences and Survival Analysis: Differences in NR3C1 expression differences between TGM2 risk groups were assessed. Survival analysis and survival curve generation were performed based on NR3C1 expression and TCGA-LUSC patient survival data. (3) Binding Sites Prediction: The JASPAR database was used to predict NR3C1 binding motifs within the TGM2 gene sequence.

To validate the direct transcriptional regulation of TGM2 by NR3C1, a chromatin immunoprecipitation (ChIP) assay was performed using the SimpleChIP^®^ Enzymatic Chromatin IP Kit (Agarose Beads) (#9002, CST, USA). Briefly, cross-linked chromatin was sonicated to generate appropriately sized fragments, followed by immunoprecipitation with an NR3C1 antibody, with IgG serving as a negative control. The enriched DNA fragments were quantitatively analyzed by qRT-PCR. Primer sequences used for ChIP-PCR are listed in **Supplementary Table S1**.

### 2.8 Statistical analysis

All *in vitro* phenotypic assays were conducted with at least three independent biological replicates per experimental condition. Data analysis and visualization were performed using R 4.3.1 and GraphPad Prism 9.0.0 (San Diego, California, USA). Band intensities were quantified using ImageJ software (v1.53t, NIH), normalized to GAPDH. Unless otherwise stated, default parameters were applied for the software packages mentioned, and data visualization was performed using the ggplot2 package (version 3.3.5). Continuous variables are presented as mean ± standard deviation (SD). Comparisons between two groups were made using the Student’s t-test or Mann-Whitney test, depending on the assumptions of homogeneity of variance and normal distribution. For comparisons among multiple groups, ordinary one-way Analysis of Variance (ANOVA) with multiple comparisons was used, except for the CCK-8 assay, which employed two-way ANOVA with multiple comparisons. Correlation analysis between two groups was conducted using the Spearman’s test, and survival analysis was performed using the Log-rank test. A P-value <0.05 was considered statistically significant, with the following notation: * (P < 0.05), ** (P < 0.01), *** (P < 0.001), and **** (P < 0.0001).

## 3 Results

### 3.1 TGM2 was an independent prognostic factor in LUSC

**Figure 1:**
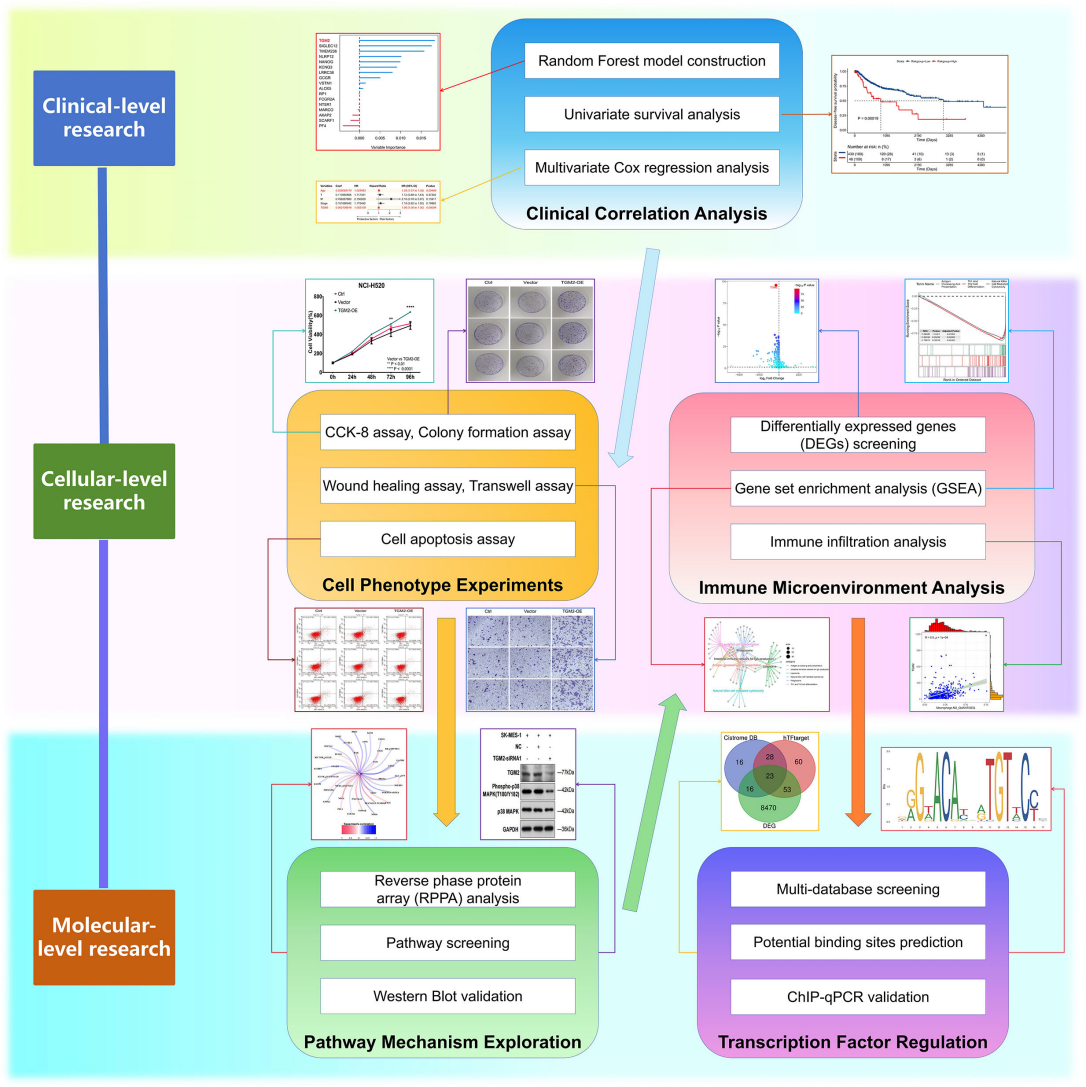
Flowchart of this study. This integrated schematic outlines the multidisciplinary approach employed in this study: Clinical-level investigations (random forest modeling, univariate/multivariate survival analysis) identified key prognostic markers; cellular phenotyping (CCK-8, colony formation, wound healing, Transwell, apoptosis assays) characterized malignant behaviors; pathway mechanisms were explored through proteomic profiling (reverse-phase protein array), targeted screening, and Western blot validation; immune microenvironment was assessed via transcriptomic analyses (differentially expressed genes, gene set enrichment, immune infiltration); preliminary exploration of transcriptional regulation involved multi-database TF screening, binding site prediction, and ChIP-qPCR experimental validation.

**Figure 2:**
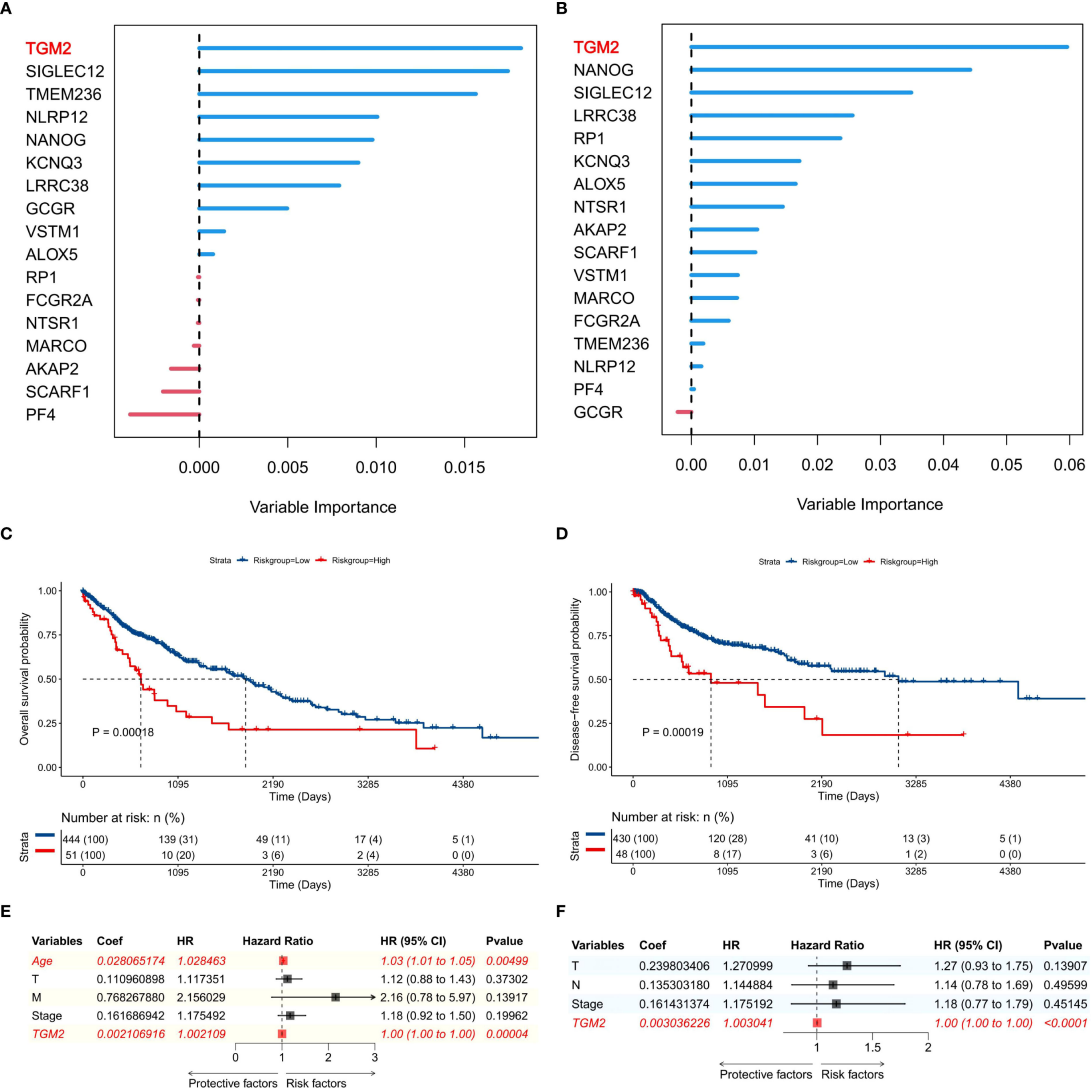
TGM2 was the top prognostic gene in the immune phenotype scoring model. **(A, B)** Importance ranking of 17 genes for predicting **(A)** overall survival (OS) and **(B)** disease-free survival (DFS) in TCGA-LUSC patients, generated by random forest analysis using the randomForestSRC package (version 3.2.3). **(C, D)** Kaplan-Meier survival curves stratified by TGM2 expression levels (TPM = 210.89) for **(C)** OS and **(D)** DFS in TCGA-LUSC patients, with statistical significance assessed by the log-rank test using the survival package (version 3.5-7) and visualized by the survminer package (version 0.4.9). **(E, F)** Forest plots showing results of multivariate Cox regression analysis for factors influencing **(E)** OS and **(F)** DFS in TCGA-LUSC patients, created using the forestploter package (version 1.1.2).

The flowchart of this study is shown in **Figure 1**. The previously developed immune phenotype scoring model (13) was reconstructed and analyzed using the random forest method. Among the 17 modeling genes, TGM2 was identified as the most important predictor for both OS (**Figure 2A**) and DFS (**Figure 2B**). Univariate survival analysis revealed that the high TGM2 expression group had significantly shorter OS (P = 0.00018, **Figure 2C**) and DFS (P = 0.00019, **Figure 2D**) compared to the low-expression group. Multivariate Cox regression analysis confirmed that TGM2 served as an independent prognostic factor for both OS (**Figure 2E**) and DFS (**Figure 2F**) in patients with LUSC. These results suggest TGM2 as a promising candidate for further investigation.

### 3.2 TGM2 exhibited oncogenic properties in LUSC

**Figure 3:**
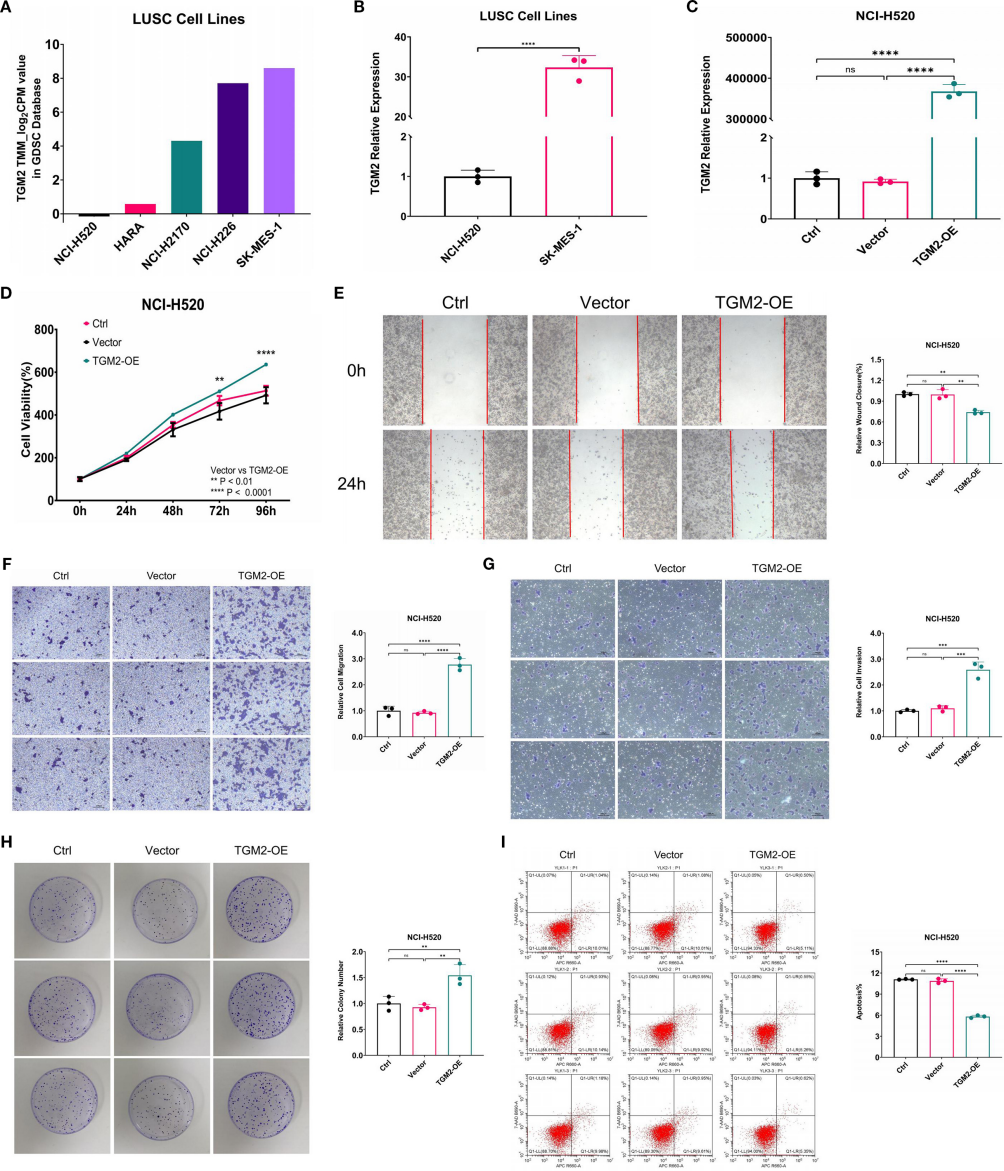
Overexpression of TGM2 in LUSC NCI-H520 cells enhanced malignant potential. **(A)** Bar plot showing TGM2 expression levels in five LUSC cell lines. **(B)** Bar plot comparing TGM2 expression between SK-MES-1 and NCI-H520 cell lines, analyzed by unpaired Student’s t-test. **(C-I)**. Comparisons among TGM2-OE, negative control (vector), and blank control (Ctrl) groups in NCI-H520 cells: **(C)** TGM2 expression, **(D)** CCK-8 assay, **(E)** wound healing assay, **(F)** Transwell migration, **(G)** Transwell invasion, **(H)** colony formation, and **(I)** apoptosis assay. Data are presented as mean ± SD from at least three independent experiments. For all assays except CCK-8, multiple group comparisons were performed using ordinary one-way ANOVA, with *post-hoc* pairwise comparisons conducted using Tukey’s multiple comparison test. For the CCK-8 assay, statistical analysis was carried out using two-way ANOVA, with Tukey’s multiple comparisons test for within-group pairwise comparisons. **P < 0.01, ***P < 0.001, ****P < 0.0001. ns, not statistically significant.

In functional studies of LUSC cells, five LUSC cell lines (NCI-H520, HARA, NCI-H2170, NCI-H226, SK-MES-1) were selected from the GDSC database, covering a wide range of endogenous TGM2 expression levels (high/medium/low), and based on commercial availability with authenticated short tandem repeat (STR) profiles. Among these, the NCI-H520 cell line exhibited the lowest TGM2 expression level, while SK-MES-1 showed the highest TGM2 expression (**Figure 3A**). Experimental validation confirmed a statistically significant difference in TGM2 expression between these two cell lines (P < 0.0001, **Figure 3B**). Consequently, SK-MES-1 was chosen for TGM2 knockdown experiments to assess loss-of-function effects, while NCI-H520 used for TGM2 overexpression studies to examine gain-of-function outcomes.

Lentiviral transfection successfully generated stable TGM2-OE NCI-H520 cells, exhibiting a more than 30,000-fold increase in TGM2 expression compared to the NC group (P < 0.0001, **Figure 3C**). Functional assays showed that TGM2 overexpression significantly enhanced malignant phenotypes in NCI-H520 cells: (1) Proliferation: The CCK-8 assay revealed significantly higher proliferation rates in the TGM2-OE group at 72 hours compared to controls (P = 0.0021, **Figure 3D**). (2) Migration and Invasion: The wound healing assay showed a 1.3443-fold increase in migration capacity (P = 0.0013, **Figure 3E**). Transwell assays revealed a 3.0107-fold increase in migrated cells (**Figure 3F**) and a 2.3543-fold increase in invaded cells (**Figure 3G**). (3) Colony Formation: The colony formation assay showed a 1.6625-fold increase in efficiency in the TGM2-OE group (P = 0.0050, **Figure 3H**). (4) Apoptosis: Flow cytometry analysis showed a 0.5333 reduction in the apoptosis rate in the TGM2-OE group (P < 0.0001, **Figure 3I**).

**Figure 4:**
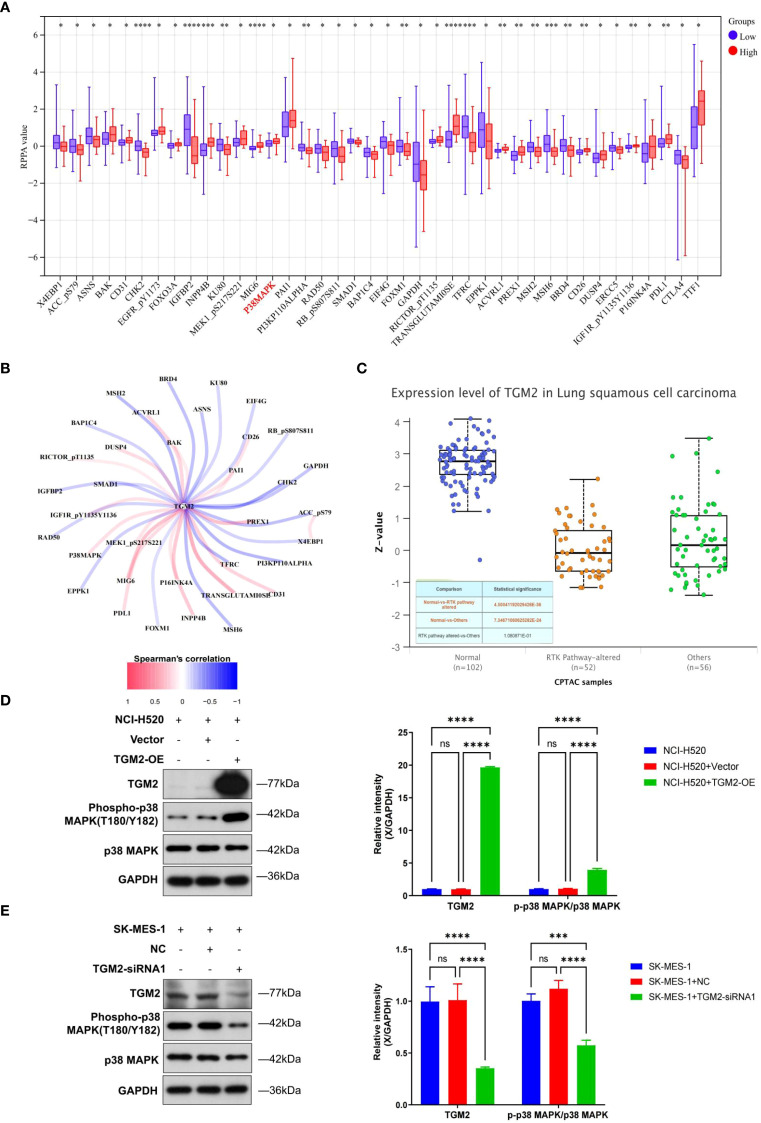
TGM2 activated the p38 MAPK signaling pathway. **(A)** Box plots showing differentially expressed proteins (DEPs) between TGM2 high- and low-risk groups through RPPA analysis; comparisons were made using the Mann-Whitney test. **(B)** Line graph showing the correlation between DEPs and TGM2 expression levels. **(C)** Box plot comparing TGM2 protein expression in receptor tyrosine kinase pathway-altered groups versus normal groups through UALCAN database analysis. D-E. Representative western blots (left) and quantitative analysis (right) of TGM2, p38 MAPK, and phosphorylated p38 MAPK in **(D)** TGM2-OE NCI-H520 cells and **(E)** TGM2-silenced SK-MES-1 cells compared with their respective negative control and blank control groups. Band intensities were quantified using ImageJ software (v1.53t, NIH), normalized to GAPDH, and multiple group comparisons were performed using two-way ANOVA and normalized to GAPDH. Data were analyzed by two-way ANOVA followed by Tukey’s multiple comparison test. *P < 0.05, **P < 0.01, ***P < 0.001, ****P < 0.0001. ns, not statistically significant.

Notably, TGM2 knockdown in SK-MES-1 cells (**Supplementary Figure S1**) produced phenotypic effects opposite to those observed in the overexpression experiments. Three siRNAs were validated for mRNA knockdown efficacy (**Supplementary Figure S1A**), and siRNA-1, selected for its balance of efficacy and specificity, was used in all functional assays, with protein-level silencing confirmed in **Figure 4E**. TGM2 knockdown in SK-MES-1 cells significantly suppressed proliferation (**Supplementary Figure S1B**), migration (**Supplementary Figures S1C, D**), invasion (**Supplementary Figure S1E**), and colony formation (**Supplementary Figure S1F**), while promoting apoptosis (**Supplementary Figure S1G**), confirming TGM2’s oncogenic role. These findings are consistent with the survival analysis results presented in **Figure 2**. Collectively, this study demonstrates that TGM2 promotes LUSC progression by enhancing proliferation, migration, invasion, and colony formation, while suppressing apoptosis.

### 3.3 TGM2 activates the p38 MAPK signaling pathway

To investigate the regulatory mechanisms of TGM2 on its downstream pathways, patients were stratified into high- and low-risk groups based on the optimal cutoff value of TGM2 expression level (TPM = 210.89), which was significantly associated with OS (**Figure 2C**), followed by proteomic analysis. RPPA analysis identified 42 DEPs between the two groups (**Figure 4A**), of which 35 exhibited significant correlations with TGM2 expression (**Figure 4B**). Notably, the protein levels of p38 MAPK and PD-L1 were significantly upregulated in the TGM2 high-risk group, while CTLA-4 was notably downregulated. Although several proteins (e.g., ACVRL1, DUSP4, PREX1) showed increased expression in the high-risk group, PD-L1 and p38 MAPK were prioritized for mechanistic validation due to their established roles in tumor-immune interactions (34, 35). This targeted approach enabled robust experimental confirmation of the TGM2-p38 MAPK regulatory axis in LUSC pathogenesis, while the complete DEPs profile serves as a resource for future investigations into additional candidates. To further explore the potential mechanisms of TGM2 in LUSC, the relationship between TGM2 expression and signaling pathways was analyzed using the UALCAN database. Statistically significant differences were observed in receptor tyrosine kinases (RTKs)-related pathways between LUSC and normal tissues (**Figure 4C**). Experimental validation confirmed that TGM2-OE NCI-H520 cells exhibited significantly higher levels of phosphorylated p38 MAPK proteins (P < 0.0001 vs. NC, **Figure 4D**), whereas TGM2-silenced SK-MES-1 cells showed marked reductions (P<0.0001 vs. NC, **Figure 4E**). These results suggest that TGM2 deficiency activates the p38 MAPK signaling pathway, thereby promoting tumor progression.

### 3.4 TGM2 may participate in the formation of an immunosuppressive microenvironment in LUSC

**Figure 5:**
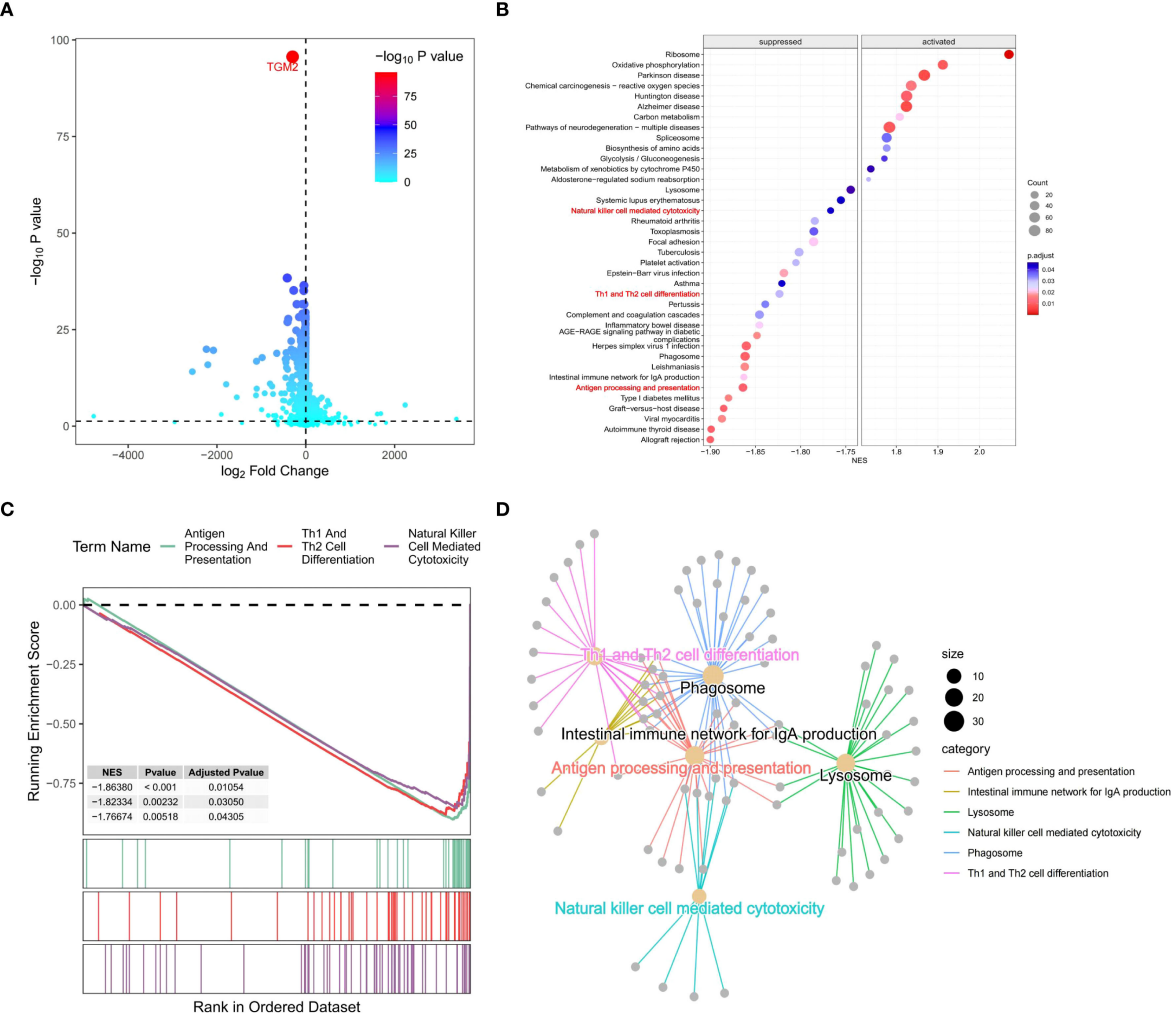
TGM2 negatively regulated immune processes in LUSC. **(A)** Volcano plot of DEGs between TGM2 risk groups generated using the ggVolcano package (version 0.0.2). **(B)** Bubble plot of GSEA results ranked by adjusted P-values, created with the GseaVis package (version 0.0.9). **(C)** Gene network diagram illustrating interactions among antigen processing and presentation, NK cell-mediated cytotoxicity, and Th1/Th2 cell differentiation pathways generated by the GseaVis package (version 0.0.9). **(D)** GSEA results for the three key pathways generated using the enrichplot package (version 1.20.3).

Samples were stratified based on TGM2 expression levels and DEGs analysis identified a total of 8,562 DEGs. Specifically, compared to the TGM2 high-risk group, 3,714 genes were significantly downregulated, while 4,848 genes were upregulated in the low-risk group (**Figure 5A**). These DEGs, along with their log_2_FC values, were subjected to GSEA, which revealed significant enrichment in 42 potential functional pathways based on adjusted P-values. The top-ranked pathways are visually highlighted im **Figure 5B**. Notably, pathways such as antigen processing and presentation (35), natural killer (NK) cell-mediated cytotoxicity (36), and Th1/Th2 cell differentiation (37) were of particular interest. To further investigate these pathways, a gene network was constructed (**Figure 5C**) and the GSEA results for these three key pathways were detailed (**Figure 5D**). Notably, all these pathways were closely associated with the p38 MAPK signaling pathway (34–37). This led to the hypothesis that TGM2 promotes LUSC progression by activating the p38 MAPK signaling pathway, while potentially exerting immunosuppressive effects *via* modulation of this pathway.

**Figure 6:**
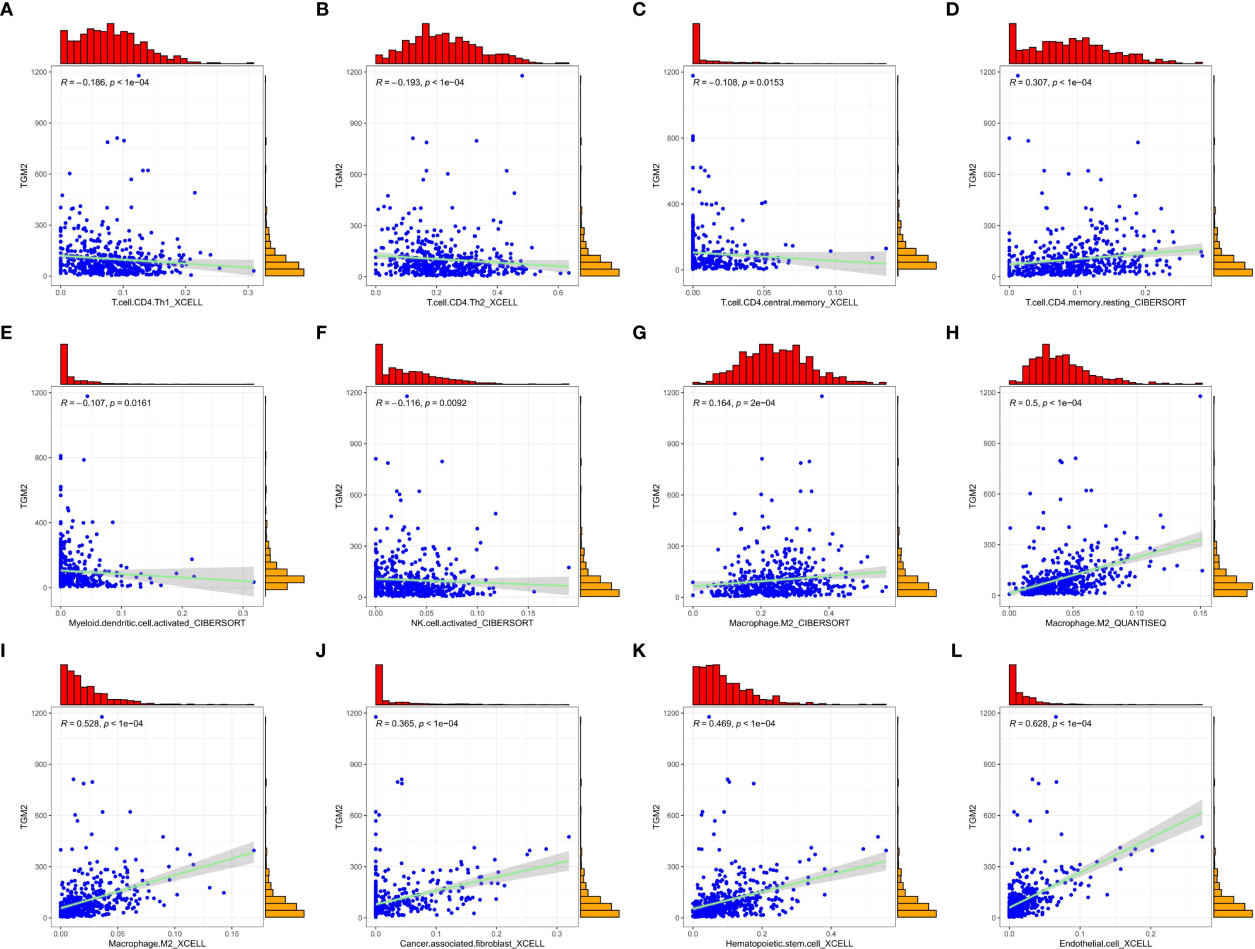
TGM2 may play a pivotal role in immune regulation in LUSC. Scatter plots demonstrate the correlations between TGM2 expression and immune cell infiltration scores. All plots were generated using the ggplot2 (version 3.5.2) and ggExtra (version 0.10.1) packages. The immune cells are as follows: **(A)** Th1 cells, **(B)** Th2 cells, **(C)** CD4^+^ central memory T cells, **(D)** resting CD4^+^ memory T cells, **(E)** activated myeloid dendritic cells, **(F)** activated NK cells, **(G-I)** M2 macrophage scores assessed by different methods, **(J)** cancer-associated fibroblasts, **(K)** hematopoietic stem cells, and **(L)** endothelial cells.

The regulatory role of TGM2 in immune cell populations, particularly CD4^+^ T cells and NK cells, was further explored by analyzing correlations between TGM2 TPM values and immune cell infiltration scores. TGM2 expression was significantly negatively correlated with Th1 cell scores (R = -0.186, P < 0.0001; **Figure 6A**), Th2 cell scores (R = -0.193, P < 0.0001; **Figure 6B**), and CD4^+^ central memory T cell scores (R = -0.108, P = 0.0153; **Figure 6C**). In contrast, TGM2 expression showed a positive correlation with resting CD4^+^ memory T cell scores (R = 0.307, P < 0.0001; **Figure 6D**). Additionally, TGM2 expression negatively correlated with activated myeloid dendritic cell scores (R = -0.107, P = 0.0161; **Figure 6E**) and NK cell activation scores (R = -0.116, P = 0.0092; **Figure 6F**). Conversely, TGM2 exhibited positive correlations with immunosuppressive cell populations, including M2 macrophages (**Figures 6G-I**), cancer-associated fibroblasts (R = 0.365, P < 0.0001; **Figure 6J**), hematopoietic stem cells (R = 0.469, P < 0.0001; **Figure 6K**), and endothelial cells (R = 0.628, P < 0.0001; **Figure 6L**). These results suggest that TGM2 plays a critical role in immune regulation, potentially influencing antigen presentation, CD4^+^ T cell and NK cell activity, and contributing to the formation of an immunosuppressive microenvironment.

To further validate the immunomodulatory role of TGM2, infiltration scores between TGM2 high- and low-risk groups were compared. TGM2-high tumors showed significant reductions in Th1 cells (P = 0.0048) and Th2 cells (P = 0.002), with concurrent increases in M2 macrophages (QUANTISEQ/XCELL, P < 0.0001), cancer-associated fibroblasts (P < 0.0001), hematopoietic stem cells (P < 0.0001), and endothelial cells (P < 0.0001) (**Supplementary Figure S2**). These findings align with our correlation analyses (**Figure 6**) and support the role of TGM2 in driving immunosuppression. Although activated NK cell infiltration did not show a significant difference (P = 0.75), the suppression of NK-mediated cytotoxicity pathways identified by GSEA (**Figures 5B–D**) suggests that TGM2 may impair NK cell function rather than abundance, potentially through p38 MAPK-mediated immunomodulation.

### 3.5 TGM2 expression was regulated by the transcription factor NR3C1

**Figure 7:**
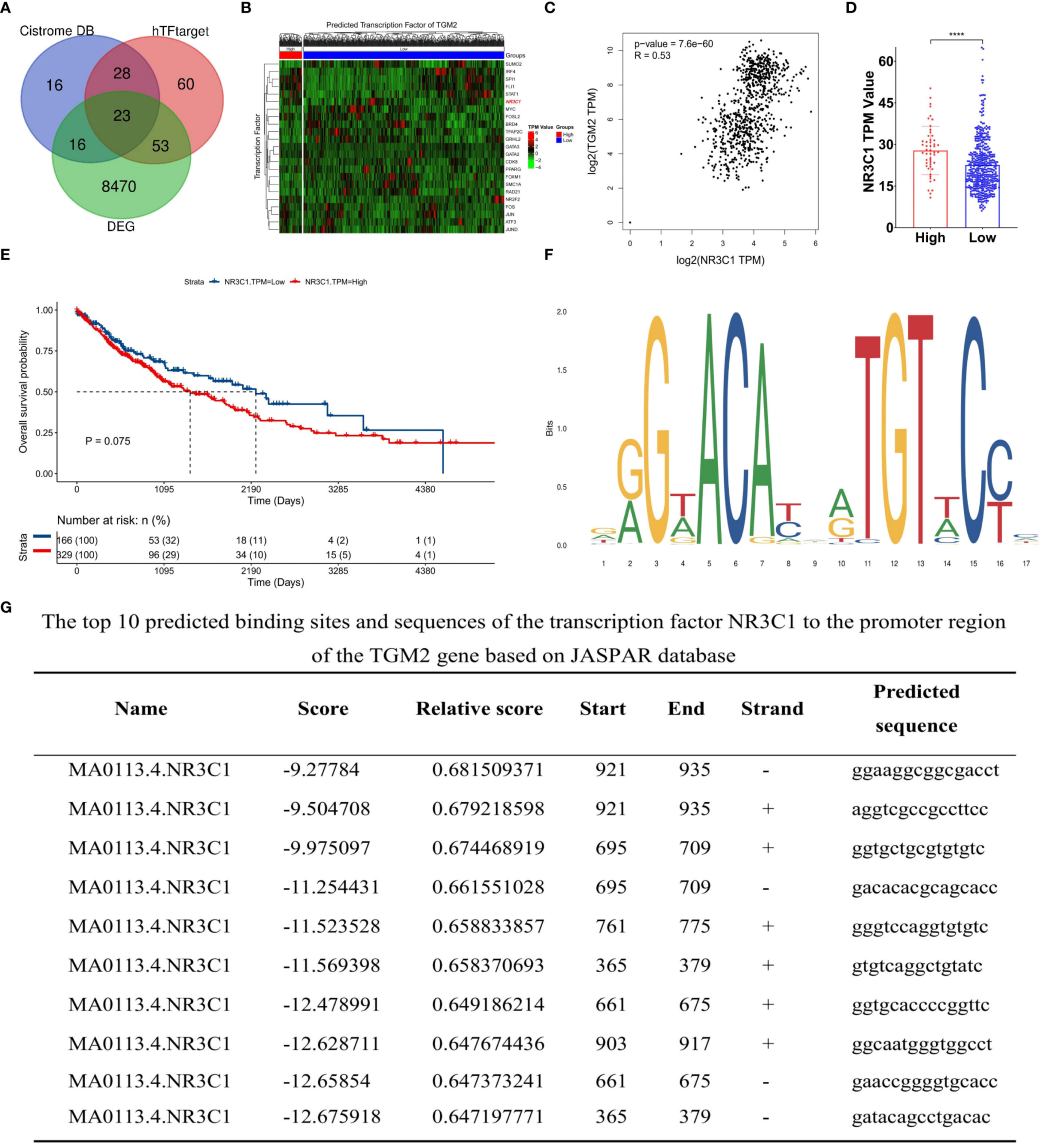
TGM2 expression may be regulated by the transcription factor NR3C1. **(A)** Venn diagram of transcription factors (TFs) predicted by Cistrome DB and hTFtarget databases to regulate TGM2 and overlapping DEGs. **(B)** Heatmap showing expression profiles of 23 key TFs in LUSC. **(C)** Scatter plot of the correlation between NR3C1 and TGM2 expression in LUSC and normal tissue. **(D)** Bar plot comparing NR3C1 expression between TGM2 high- and low-risk groups. **(E)** Survival analysis of TCGA-LUSC patients stratified by NR3C1 expression (optimal cutoff: TPM = 17.73). **(F)** Nucleotide sequence features of NR3C1 binding regions predicted by JASPAR. **(G)** Top 10 predicted NR3C1 binding sites and sequences in the TGM2 promoter region. ****P < 0.0001.

To explore the direct regulatory mechanisms of the TGM2 gene, its upstream regulators, particularly TFs, were identified. The Cistrome DB and hTFtarget databases were employed to predict potential TFs regulating TGM2, resulting in 23 key candidates identified through intersection analysis with DEGs (**Figure 7A**). The expression profiles of these TFs in LUSC are shown in **Figure 7B**. Based on Cistrome DB prediction scores, NR3C1, the top-ranked TF, was prioritized for further investigation. Analysis using the GEPIA database revealed a significant positive correlation between TGM2 and NR3C1 expression in both normal and tumor tissues (R = 0.53, P = 7.6×10^−60^; **Figure 7C**). Furthermore, NR3C1 expression significantly differed between TGM2 high- and low-risk groups (P < 0.0001; **Figure 7D**). Although survival analysis did not reach statistical significance, there was a trend suggesting poorer prognosis for patients with LUSC exhibiting high NR3C1 expression (P = 0.075; **Figure 7E**). To investigate potential direct regulatory interactions, NR3C1 binding sites in the TGM2 promoter region were predicted using the JASPAR database. **Figure 7F** illustrates the nucleotide sequence features of NR3C1 binding regions, and **Figure 7G** lists the top ten predicted binding sites and their sequences. These findings laid the groundwork for further exploration of TGM2’s regulatory mechanisms.

**Figure 8:**
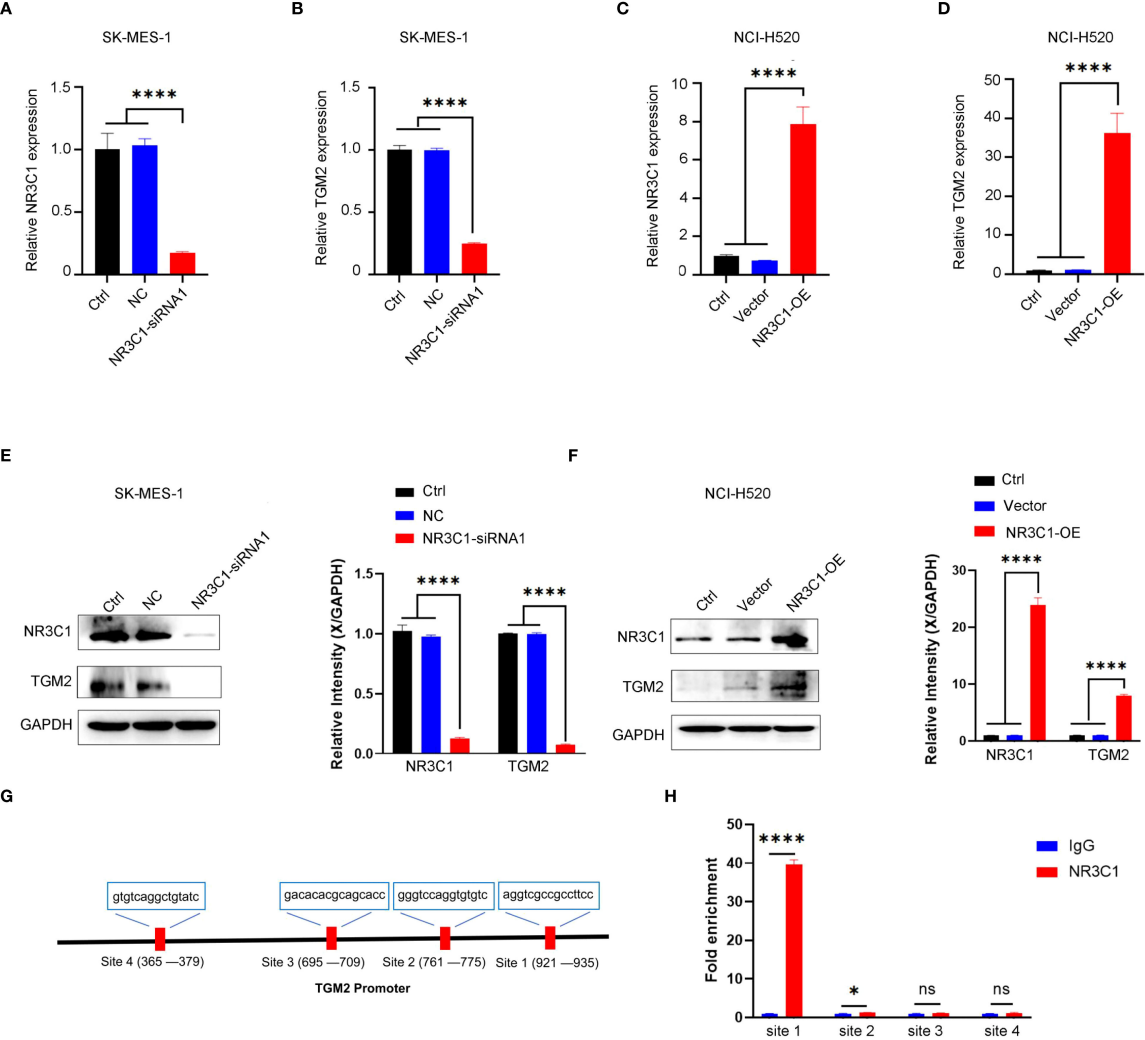
NR3C1 transcriptionally regulates TGM2 expression in LUSC. A-B. NR3C1 knockdown in SK-MES-1 cells. **(A)** mRNA expression of NR3C1 and **(B)** TGM2 was measured by qRT-PCR. siRNA-1, which demonstrated the highest knockdown efficacy, was selected for all subsequent functional experiments. C-D. NR3C1 overexpression in NCI-H520 cells. **(C)** mRNA expression of NR3C1 and **(D)** TGM2 was measured by qRT-PCR. E-F. Western blot analysis of NR3C1 and TGM2 protein levels in **(E)** NR3C1-knockdown and **(F)** NR3C1-overexpressing cells. GAPDH served as a loading control. Band intensities were quantified using ImageJ and normalized to GAPDH. **(G)** Schematic diagram of the TGM2 promoter region showing the locations of four predicted NR3C1 binding sites (Site 1: 921–935; Site 2: 1050–1064; Site 3: 1120–1134; Site 4: 1250–1264). **(H)** ChIP-qPCR validation of NR3C1 binding to the TGM2 promoter. Site 1 showed the strongest enrichment, indicating it as the primary binding site. IgG was used as a negative control. Statistical analysis was performed using ordinary one-way ANOVA **(A–D)** or two-way ANOVA **(E, F)** with Tukey’s multiple comparisons test or unpaired Student’s t-test **(H)**. *P < 0.05, ****P < 0.0001. ns, not statistically significant.

To assess the regulatory role of NR3C1, two distinct siRNAs were used for knockdown experiments (**Supplementary Figure S3**). siRNA1, selected for its efficacy, was used in subsequent functional studies, demonstrating that NR3C1 knockdown led to a concomitant decrease in TGM2 mRNA levels (**Figures 8A, B**). Conversely, NR3C1 overexpression resulted in the opposite effect, increasing TGM2 mRNA expression (**Figures 8C, D**), suggesting that NR3C1 promotes TGM2 transcription. Western blot analysis further confirmed that NR3C1 knockdown reduced TGM2 protein levels, while NR3C1 overexpression enhanced them (**Figures 8E, F**), indicating that NR3C1 upregulates TGM2 protein expression likely by enhancing its transcription. To directly test whether NR3C1 promotes TGM2 transcription, ChIP assays were performed targeting four predicted NR3C1 binding sequences (selected based on a prediction score threshold of >0.65, as shown in **Figure 7G**) within the TGM2 promoter region. The locations of the four validated sequences are shown in **Figure 8G**. The results identified Site 1 (921-935) as the most probable NR3C1 binding site on the TGM2 promoter (**Figure 8H**), suggesting that NR3C1 likely binds to Site 1 (921-935) to stimulate TGM2 transcription, thereby upregulating TGM2 protein expression. In summary, NR3C1 was identified as a potential upstream transcriptional regulator of TGM2, and their expression correlation and direct regulatory relationship were preliminarily revealed.

**Figure 9:**
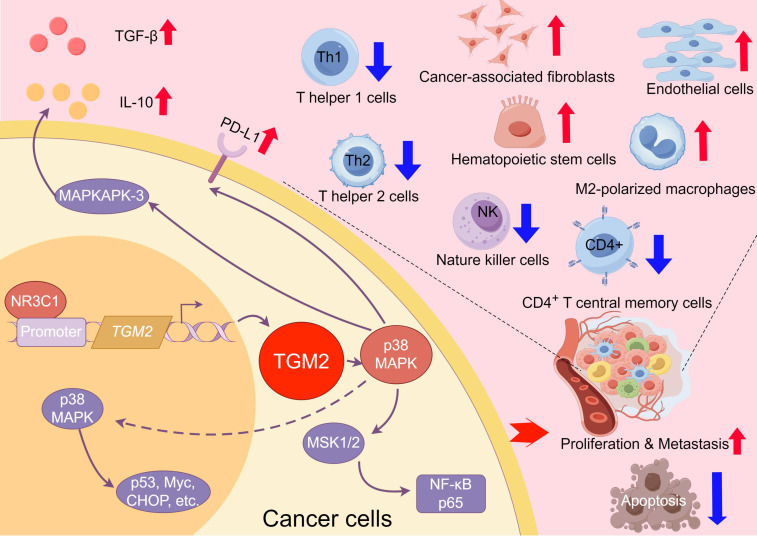
Schematic diagram of the TGM2/NR3C1-p38 MAPK axis driving tumor progression and immune evasion in LUSC. This figure illustrates the key mechanisms of TGM2 in LUSC. The transcription factor NR3C1 binds to the promoter region of the TGM2 gene to regulate its expression. Elevated TGM2 activates the p38 MAPK signaling pathway, promoting tumor cell proliferation, metastasis, and apoptosis resistance, thereby accelerating tumor progression. Additionally, TGM2 may reshape the tumor microenvironment by modulating the p38 MAPK pathway, including shaping an immunosuppressive microenvironment, upregulating immune checkpoint molecules, promoting secretion of immunosuppressive factors, and enhancing immune evasion. This study is the first to elucidate the dual role of the NR3C1/TGM2-p38 MAPK axis in driving tumor malignancy and immune microenvironment imbalance, providing mechanistic insights that could support the future development of targeted-immunotherapy combination strategies in LUSC.

These findings deepen our understanding of the role of TGM2 in LUSC pathogenesis, provide directions for future research and offer valuable insights for developing potential therapeutic strategies targeting TGM2 and its regulatory network. The schematic diagram of the scientific hypothesis in this study is shown in **Figure 9**, created using Figdraw (https://www.figdraw.com).

## 4 Disscussion

In the context of lung cancer, two cohort studies involving patients with NSCLC have identified TGM2 as a crucial biomarker associated with increased tumor invasiveness, metastatic potential, and poor prognosis (38, 39). A study in a Korean patient cohort demonstrated that TGM2 upregulation was significantly linked to shorter DFS in non-LUAD subtypes (38), while another study in a Chinese cohort found similar associations in both non-LUAD and LUAD subtypes (39). To further elucidate the role of TGM2 in lung cancer, extensive *in vitro* studies have shown its involvement in regulating cell invasion, migration, and drug sensitivity. For example, Lee et al. (40) demonstrated that TGM2 promotes lung cancer cell migration and invasion through a mechanism independent of its transamidase activity, using the weakly invasive LUAD cell line CL1-0 and its highly invasive counterpart CL1-5. Additionally, TGM2 has been shown to enhance radioresistance, promote DNA repair, and interact directly with TOP2 in LUAD cells. Reduced TGM2 expression induces oxidative stress and triggers p53-independent extrinsic and intrinsic apoptosis (41). In the present study, systematic bioinformatics analyses revealed TGM2 as an independent prognostic factor in LUSC. Specifically, patients with high TGM2 expression exhibited significantly shorter OS and DFS compared to those with low expression, emphasizing the clinical relevance of TGM2 in LUSC prognosis. Collectively, investigating the functional mechanisms of TGM2 has significant clinical implications for improving lung cancer diagnosis and treatment efficacy, as well as prolonging patient survival.

The MAPK signaling pathway is a crucial oncogenic cascade that transmits extracellular signals from cell surface receptors, primarily RTKs, to downstream effectors such as RAS, RAF, MEK, and ERK, thereby promoting cell survival, growth, and differentiation (42–44). Oncogenic mutations in key MAPK components drive up to 30% of human cancers, particularly NSCLC, which is frequently initiated by mutually exclusive mutations in RTKs such as EGFR, HER2, ALK, ROS1, KRAS, and BRAF (45–48). In this study, a correlation between TGM2 expression and RTK alterations was identified. Experimental validation further confirmed that TGM2 enhances LUSC cell proliferation, metastasis, and apoptosis resistance by activating the p38 MAPK pathway, thus augmenting the malignant potential. Notably, MEK inhibition effectively suppresses ERK hyperactivation, inhibiting tumor growth and survival (49–51). While MEK mutations are rare in human tumors, aberrant MEK activity—driven by upstream RAS or RAF mutations—is observed in over 85% (52) of cancers, making MEK an attractive therapeutic target. Clinical trials have demonstrated the efficacy of MEK inhibitors in NSCLC, particularly when combined with chemotherapy or other agents. For example, the FDA-approved combination of MEK and BRAF inhibitors effectively halts tumor progression in KRAS/BRAF-mutant patients with NSCLC (52–54). Therefore, MEK inhibitors may offer therapeutic potential for patients with LUSC exhibiting high TGM2 expression.

Activation of the MAPK pathway not only drives tumor progression but also induces the expression of immunosuppressive molecules such as PD-L1, establishing a vicious cycle of immune evasion (55–58). In lung cancer, activation of the FGFR1/MAPK axis regulates PD-L1 expression, suppresses CD8^+^ and CD3^+^ T cell infiltration, and promotes immunosuppression (56). In hepatocellular carcinoma, the EGFR-p38 MAPK axis enhances PD-L1 expression *via* miR-675-5p and downregulates human leukocyte antigen class-I (HLA-I), impairing immune regulation (57). In melanoma, inhibition of the MAPK pathway correlates with resistance to ICIs, while MEK inhibitor resistance is associated with to loss of MHC-I antigen presentation, reduced T cell infiltration, and poor clinical responses to anti-PD-1 therapy (58). These mechanisms have prompted preclinical studies on the synergistic effects of MEK inhibitors and PD-1/PD-L1 blockade. Combined therapy enhances antitumor immune responses and reverses immunosuppressive microenvironments by modulating PD-L1 expression (59–61). For instance, the MEK inhibitor selumetinib increases PD-1 expression on CD8^+^ T cells in KRAS-mutant NSCLC (59), while dual inhibition of MEK and PD-1/PD-L1 elevates tumor-infiltrating CD8^+^ and CD4^+^ T cells in KRAS/p53-driven lung cancer models (60). Notably, combining MEK inhibitors with PD-L1 antibodies induces synergistic tumor regression in mouse models, providing a rationale for combination therapy in advanced NSCLC (61). These studies collectively suggest that targeting the MAPK pathway may rectify immune dysfunction and remodel the TME. Our findings indicate that the TGM2-MAPK axis in LUSC may impair antigen presentation, hinder CD4^+^ T cell activation, and elevate immunosuppressive populations (**Figure 6**). Grouped comparisons confirmed reduced Th1/Th2 cells and an expanded immunosuppressive stroma in TGM2-high tumors (**Supplementary Figure S2**), mechanistically linking TGM2 to impaired anti-tumor immunity. Thus, TGM2-mediated MAPK activation may directly suppress innate immune cell function and remodel immune checkpoint networks through upstream signaling cascades, warranting further investigation.

Regarding NK cells, GSEA (**Figures 5B–D**) revealed significant enrichment of the NK cell-mediated cytotoxicity pathway in TGM2-low tumors, indicating functional impairment of NK cell activity within TGM2-high microenvironments. Although the grouped comparison of activated NK cells (CIBERSORT) did not reach statistical significance (P = 0.75), this aligns with the relatively weak negative correlation (R = -0.116, P = 0.0092) observed in **Figure 6F**. This suggests: (1) The suppression at the pathway level likely reflects impaired NK cell function (e.g., cytotoxicity, cytokine production) rather than a reduction in absolute cell number; (2) Technical limitations of bulk deconvolution algorithms may obscure subtle functional states; and (3) TGM2 may influence NK cell activity indirectly through p38 MAPK-driven immunosuppressive factors (e.g., TGF-β, IL-10), which could affect function without altering infiltration scores. This interpretation is supported by previous studies linking p38 MAPK to NK cell dysfunction (34–37).

In exploring the regulatory mechanisms of TGM2, NR3C1 was identified as a potential upstream regulator. Our study revealed a significant positive correlation between TGM2 and NR3C1 expression, with a trend toward poorer prognosis in patients with LUSC exhibiting high NR3C1 expression. Previous research has implicated NR3C1 in regulating inflammation (62, 63), autophagy, immune cell modulation (64), cell growth (64, 65), ferroptosis (66), and glucocorticoid-induced apoptosis (67). In breast cancer, NR3C1 expression is associated with NF-κB pathway activation, influencing cancer cell proliferation and migration (65). In gastric cancer, NR3C1 overexpression correlates with resistance to 5-fluorouracil (5-FU) (68). In ovarian cancer, NR3C1 upregulates ROR1, altering cell differentiation and chemosensitivity (69). In clear cell renal cell carcinoma, NR3C1 knockdown activates endoplasmic reticulum stress and mitophagy *via* the ATF6-PINK1/BNIP3 pathway, suppressing tumor proliferation and migration (70). Despite these insights, the role of NR3C1 in lung cancer remains largely unexplored. This study is the first to identify NR3C1 as a potential upstream regulator of TGM2 in LUSC, offering novel insights into its role in tumorigenesis and progression.

This study, through systematic bioinformatics and experimental validation, elucidates the critical role of TGM2 in LUSC and its potential link to immunotherapy efficacy. However, several limitations must be acknowledged. First, reliance on public databases may introduce bias due to limited sample size and data diversity. Future studies should expand sample cohorts and include multi-ethnic data to enhance generalizability. Second, while our *in vitro* experiments using stably transfected TGM2-OE cell lines provide mechanistic insights into the pro-tumorigenic and immunomodulatory roles of TGM2, the critical next step is validating these findings *in vivo*. Utilizing these established cell lines in appropriate animal models (e.g., xenograft or syngeneic models) is essential to confirm the role of the TGM2/NR3C1-p38 MAPK axis in tumor progression and immune evasion within a physiologically relevant microenvironment, as well as to evaluate its potential as a therapeutic target. Conducting these *in vivo* studies represents a primary focus of our ongoing and future research efforts. Third, although TIMER2.0 analysis offers a validated computational approach for inferring immune cell infiltration, its resolution is inherently limited compared to single-cell or spatial profiling techniques. Future studies employing multiplexed tissue imaging or spatial transcriptomics will be crucial for delineating the spatial dynamics of TGM2-mediated immune remodeling. Lastly, while Chang et al. (26) previously established the prognostic value of TGM2 and its association with the inflammatory TME and immunotherapy sensitivity in LUSC, our study significantly advances the field by uncovering the underlying molecular mechanisms and upstream regulation. Specifically, this study provides: (1) comprehensive functional validation using both knockdown and overexpression models, confirming the pro-tumorigenic effects of TGM2; (2) identification of the p38 MAPK signaling pathway as a critical downstream effector mediating the oncogenic and immunomodulatory functions of TGM2; and crucially, (3) the first identification of the transcription factor NR3C1 as a novel upstream regulator of TGM2 expression. Collectively, these findings uncover the previously unrecognized NR3C1/TGM2-p38 MAPK axis, offering new mechanistic insights that could inform future therapeutic strategies for targeting TGM2 in LUSC.

This study highlights the pivotal role of TGM2 in LUSC, establishing its potential as an independent prognostic marker. High TGM2 expression correlates with reduced OS and DFS, promoting tumor proliferation, migration, invasion, and resistance to apoptosis. Additionally, TGM2 exerts its oncogenic and immunomodulatory effects through p38 MAPK pathway activation, providing new mechanistic insights. As a key molecular marker in LUSC, TGM2 holds significant clinical translational value. Future research should focus on elucidating its regulatory mechanisms, particularly its interaction with NR3C1, to explore the potential for developing effective therapeutic strategies for patients with LUSC.

## Data Availability

The original contributions presented in the study are included in the article/**Supplementary Material**. Further inquiries can be directed to the corresponding authors.

## Supplementary material

Supplementary Figure 1 TGM2 knockdown in SK-MES-1 cells suppressed malignant phenotypes. **(A)** Validation of TGM2 mRNA knockdown efficiency by three different siRNAs in SK-MES-1 cells. siRNA-1 was selected for subsequent functional assays due to its balanced efficacy and specificity. **(B–G)** Phenotypic effects of TGM2 knockdown (siRNA-1) compared to negative control (NC) and blank control (Ctrl) groups: **(B)** CCK-8 proliferation assay, **(C)** wound healing assay, **(D)** Transwell migration, **(E)** Transwell invasion, **(F)** colony formation, and **(G)** apoptosis assay. TGM2 knockdown significantly suppressed proliferation, migration, invasion, and colony formation, while promoting apoptosis, providing reciprocal functional validation of its oncogenic role in LUSC. Statistical analysis was performed using ordinary one-way ANOVA **(A, C–G)** or two-way ANOVA **(B)** with Tukey’s multiple comparisons test. **P < 0.01, ***P < 0.001, ****P < 0.0001.

Supplementary Figure 2 Comparison of immune cell infiltration scores between TGM2 high- and low-risk groups. Box plots showing the differences in estimated immune cell infiltration abundances between the TGM2 high-risk and low-risk groups. Infiltration scores were derived from the TIMER2.0 database. TGM2-high tumors exhibited significantly reduced infiltration of Th1 cells (P = 0.0048) and Th2 cells (P = 0.002). Infiltration of immunosuppressive cells was significantly increased, including M2 macrophages (QUANTISEQ, P < 0.0001), M2 macrophages (XCELL, P < 0.0001), cancer-associated fibroblasts (P < 0.0001), hematopoietic stem cells (P < 0.0001), and endothelial cells (P < 0.0001). No statistically significant differences were observed in the infiltration levels of M2 macrophages (CIBERSORT, P = 0.06), (I) CD4^+^ central memory T cells (XCELL, P = 0.71), activated NK cells (CIBERSORT, P = 0.75), or activated myeloid dendritic cells (CIBERSORT, P = 0.82). Statistical significance was determined using the Mann-Whitney test. ns, not significant; *P < 0.05, **P < 0.01, ****P < 0.0001.

Supplementary Figure 3 Screening of NR3C1 siRNA and validation of knockdown efficiency. qRT-PCR analysis of NR3C1 mRNA expression in SK-MES-1 cells transfected with two independent siRNAs or negative control siRNA (NC). siRNA-1 was selected for all subsequent functional studies due to its superior knockdown efficiency. Statistical analysis was performed using ordinary one-way ANOVA with Tukey’s multiple comparisons test. ****P < 0.0001.
