# Supplementary figures and images for: TGM2 regulated by transcription factor NR3C1 drives p38 MAPK-mediated tumor progression and immune evasion in lung squamous cell carcinoma
Source: Front Immunol. 2025 Sep 18;16:1595907. doi: 10.3389/fimmu.2025.1595907 (PMC12488625; doi:10.3389/fimmu.2025.1595907)

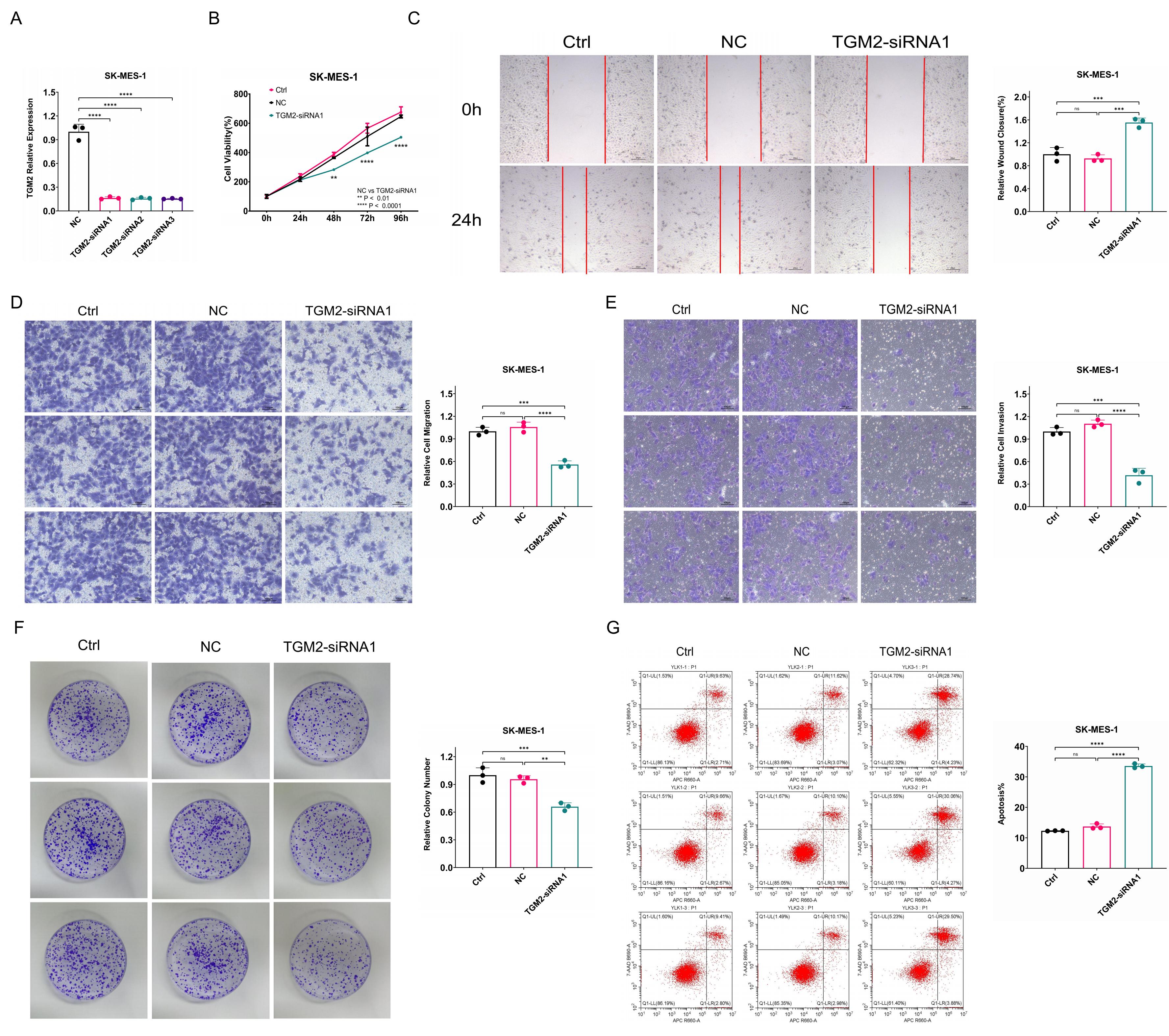

Supplement: Supplementary Figure 1 — TGM2 knockdown in SK-MES-1 cells suppressed malignant phenotypes. (A) Validation of TGM2 mRNA knockdown efficiency by three different siRNAs in SK-MES-1 cells. siRNA-1 was selected for subsequent functional assays due to its balanced efficacy and specificity. (B–G) Phenotypic effects of TGM2 knockdown (siRNA-1) compared to negative control (NC) and blank control (Ctrl) groups: (B) CCK-8 proliferation assay, (C) wound healing assay, (D) Transwell migration, (E) Transwell invasion, (F) colony formation, and (G) apoptosis assay. TGM2 knockdown significantly suppressed proliferation, migration, invasion, and colony formation, while promoting apoptosis, providing reciprocal functional validation of its oncogenic role in LUSC. Statistical analysis was performed using ordinary one-way ANOVA (A, C–G) or two-way ANOVA (B) with Tukey’s multiple comparisons test. **P < 0.01, ***P < 0.001, ****P < 0.0001. [file DataSheet1.zip › figureS1_01.jpg]

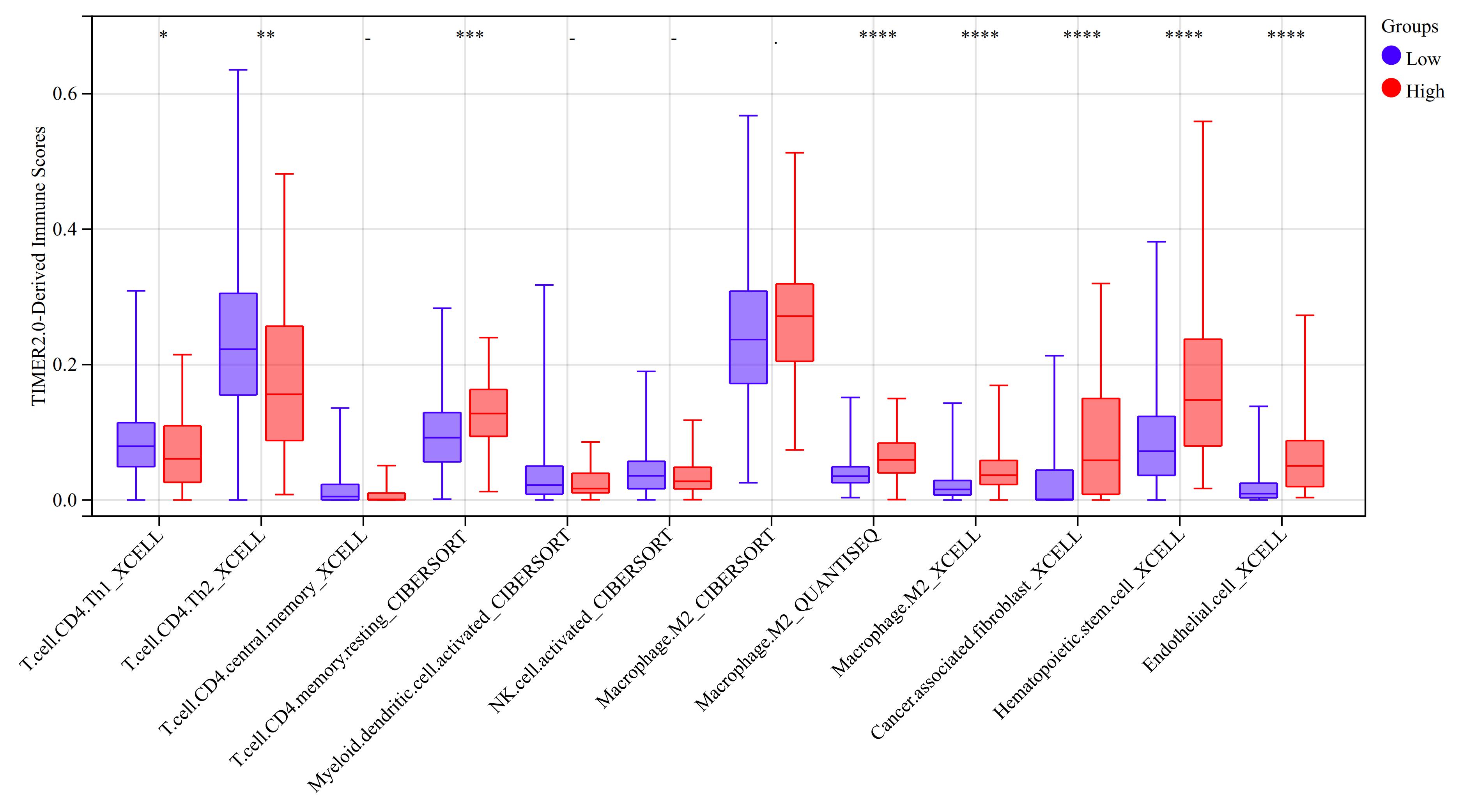

Supplement: Supplementary Figure 1 — TGM2 knockdown in SK-MES-1 cells suppressed malignant phenotypes. (A) Validation of TGM2 mRNA knockdown efficiency by three different siRNAs in SK-MES-1 cells. siRNA-1 was selected for subsequent functional assays due to its balanced efficacy and specificity. (B–G) Phenotypic effects of TGM2 knockdown (siRNA-1) compared to negative control (NC) and blank control (Ctrl) groups: (B) CCK-8 proliferation assay, (C) wound healing assay, (D) Transwell migration, (E) Transwell invasion, (F) colony formation, and (G) apoptosis assay. TGM2 knockdown significantly suppressed proliferation, migration, invasion, and colony formation, while promoting apoptosis, providing reciprocal functional validation of its oncogenic role in LUSC. Statistical analysis was performed using ordinary one-way ANOVA (A, C–G) or two-way ANOVA (B) with Tukey’s multiple comparisons test. **P < 0.01, ***P < 0.001, ****P < 0.0001. [file DataSheet1.zip › figureS2_01.jpg]

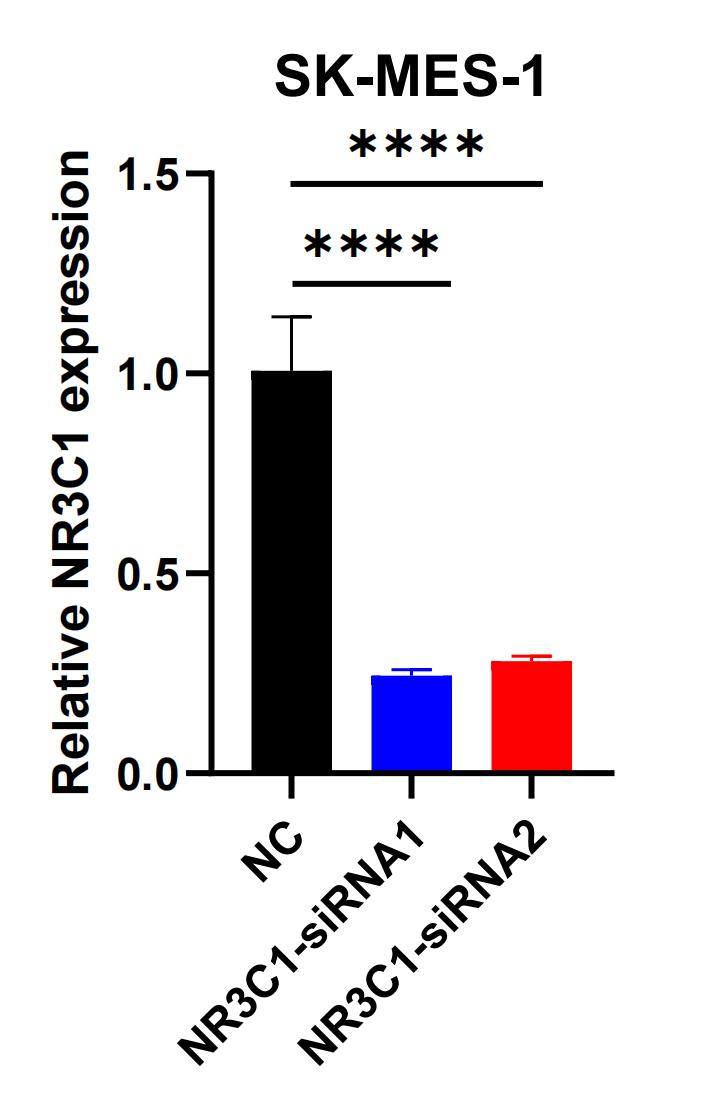

Supplement: Supplementary Figure 1 — TGM2 knockdown in SK-MES-1 cells suppressed malignant phenotypes. (A) Validation of TGM2 mRNA knockdown efficiency by three different siRNAs in SK-MES-1 cells. siRNA-1 was selected for subsequent functional assays due to its balanced efficacy and specificity. (B–G) Phenotypic effects of TGM2 knockdown (siRNA-1) compared to negative control (NC) and blank control (Ctrl) groups: (B) CCK-8 proliferation assay, (C) wound healing assay, (D) Transwell migration, (E) Transwell invasion, (F) colony formation, and (G) apoptosis assay. TGM2 knockdown significantly suppressed proliferation, migration, invasion, and colony formation, while promoting apoptosis, providing reciprocal functional validation of its oncogenic role in LUSC. Statistical analysis was performed using ordinary one-way ANOVA (A, C–G) or two-way ANOVA (B) with Tukey’s multiple comparisons test. **P < 0.01, ***P < 0.001, ****P < 0.0001. [file DataSheet1.zip › figureS3_01.jpg]
